# Articles You Might Have Missed

**DOI:** 10.1007/s13181-026-01150-8

**Published:** 2026-08-14

**Authors:** Tyler J. Yates, Kathleen Q. Chen, Zackery L. G. Fowler, Haley A. Kelly, Austin W. Gay

**Affiliations:** 1 Department of Emergency Medicine, Division of Medical Toxicology, University of Texas Southwestern Medical Center, Dallas, TX USA; 2 Parkland Memorial Hospital, Dallas, TX USA

**Keywords:** Opioid-related disorders, Paraquat, Neonatal abstinence syndrome, Coral snakes, Mitragyna

## Article #1:

Friedmann PD, Wilson D, Stopka TJ, et al. Medications for Opioid Use Disorder in County Jails: Outcomes After Release. New Engl J Med 2026:393(10):994-1003. https://doi.org/10.1056/nejmsa2415987.

### Background

Individuals with opioid use disorder (OUD) are disproportionately represented in correctional settings and face a high risk of overdose and death after release. Although methadone, buprenorphine, and naltrexone effectively treat OUD, access to these medications for opioid use disorder (MOUD) remains limited in jails in the US. In 2019, seven county jails in Massachusetts (MA) began pilot programs offering all three US Food and Drug Administration (FDA)-approved MOUD agents.

### Research Question

Is receipt of MOUD during incarceration associated with improved post-release outcomes?.

### Methods

This was a retrospective observational study using linked jail and state public health data in seven MA county jails between September 2019 and December 2020. Individuals with probable OUD received treatment with methadone, buprenorphine, naltrexone, or no MOUD while in jail, per local standard care. Primary outcomes were post-release treatment with MOUD, opioid overdose, reincarceration, and death from any cause. Propensity score weighting was used to adjust for measured baseline differences.

### Results

The cohort included 6,400 participants, of whom 2711 (42.4%) received MOUD and 3,689 (57.6%) did not. Median age was 36 years, and most subjects (74.7%) were male. Nearly 74% of treated participants were already receiving MOUD at jail entry compared to 17% of untreated participants. Buprenorphine was given most frequently (68%), followed by methadone (26%) and extended-release naltrexone (6.5%). Individuals who received MOUD in jail were more likely to continue MOUD post-release. Receipt of MOUD in jail was also associated with a significantly lower risk of fatal opioid overdose (adjusted odds ratio: 0.48; 95% confidence interval: 0.36 – 0.64), non-fatal opioid overdose (aOR 0.76; 95% CI: 0.68 – 0.85), reincarceration (aOR: 0.88; 95% CI: 0.81 – 0.94), and death from any cause (aOR: 0.44; 95% CI: 0.35 – 0.56).

### Conclusion

Receipt of MOUD during jail incarceration was associated with greater continuation of OUD treatment post-release and with lower risks of opioid overdose, reincarceration, and all-cause mortality. The findings support routine continuation and initiation of MOUD in correctional settings with strong continuity to community treatment after release.

### Critique

This study has the typical limitations inherent to its observational design, and there were substantial baseline differences between groups. Variable OUD definitions across jails, incomplete capture of out-of-state outcomes, the COVID-era study period, and limited generalizability beyond MA are additional limitations. A further concern is equability: non-white participants were less likely to receive MOUD, which may reflect existing community disparities in pre-incarceration MOUD access.

### Implication for Toxicologists

Incarceration and release represent critical opportunities for life-saving opioid overdose-prevention. Within its limitations, this study supports MOUD initiation and continuation for incarcerated persons to reduce the risk of post-release opioid misuse, reincarceration, opioid overdose, and death. Beyond short-term withdrawal control, MOUD should be understood as a mortality-reduction intervention requiring continuity across emergency, inpatient, correctional, and outpatient systems.

## Article #2:

Mohan A, Harikrishna J, Venkata Rami Reddy B, et al. Clinical Presentation, Laboratory Abnormalities, and Predictors of Outcome in 103 Patients With Acute Paraquat Poisoning: A Prospective Observational Study. Crit Care Med 2025:53(10):e2016-e2024. https://doi.org/10.1016/0148-9062(81)90514-3.

### Background

Paraquat is a highly toxic herbicide associated with substantial morbidity and mortality worldwide, particularly in low- and middle- income countries where there is widespread access. Paraquat ingestion is a common means of self-harm in countries where bans are not enforced. Despite advances in supportive care, mortality remains high due to progressive pulmonary fibrosis and multi-system organ failure caused by free radical formation. Plasma paraquat concentrations are largely unavailable in resource limited settings. This study sought to identify readily available clinical and laboratory predictors of mortality in patients with acute paraquat poisoning.

### Research Question

Among patients presenting with acute paraquat self-poisoning, which clinical and laboratory abnormalities are associated with mortality?

### Methods

This was a prospective observational study conducted at a single tertiary care medical center in India between August 2023 and March 2024. Patients with intentional single-substance ingestions of paraquat admitted to the medicine wards and medical ICU were included. Prospectively collected variables included demographic information, estimated quantity ingested, clinical manifestations, laboratory values, imaging studies, and severity scores, such as Acute Physiology and Chronic Health Evaluation II (APACHE II) and International Program on Chemical Safety Poisoning Severity Scores (ICPSS-PSS). Patients were followed through hospitalization until discharge or death. The primary outcome was in-hospital mortality. Univariate analyses identified variables associated with mortality, followed by multivariable logistic regression to determine independent predictors of death.

### Results

103 patients were enrolled, with a mean age of 29.5 years; 71% were male. In-hospital mortality was 76%. Non-survivors demonstrated larger reported ingestion volumes, higher serum creatinine and bilirubin, higher APACHE II and ICPSS-PSS scores, and lower PaO₂/FiO₂ ratios. Idealized cut-off scores for mortality were an APACHE II score greater than 10 on initial presentation (sensitivity: 91%; specificity: 72%) and IPCS-PSS scores greater than 2 on initial presentation (sensitivity: 73%; specificity: 84%). Score performance of the APACHE II and IPCS-PSS measures were not statistically different (APACHE II AUC: 0.91; IPCS-PSS AUC: 0.82). Multivariate analysis found that shortness of breath on initial presentation, hepatic dysfunction (defined as total serum bilirubin greater than 1.2 mg/dL) on initial presentation, and pulmonary infiltrates on chest imaging at any time during treatment were strong independent predictors of mortality. Longer hospital length-of-stay was independently associated with decreased mortality.

### Conclusion

Despite advances in supportive care, acute paraquat poisoning continues to have high mortality. Clinicians can use readily available clinical and laboratory findings such as dyspnea, hyperbilirubinemia, chest imaging, oxygenation indices, and APACHE II or IPCS-PSS scores to estimate the risk of mortality when quantitative paraquat assays are unavailable.

### Critique

Although observational, this study was strengthened by its prospective cohort design. The single center design limits external validity, and no information was provided about patients who were not hospitalized. Additional weaknesses include using patient self-report to estimate the amount of paraquat ingested and lack of plasma or serum confirmation or quantification. Care was not standardized or clearly described, and patients were not followed after discharge.

### Implication for Toxicologists

This article demonstrates that readily available clinical, laboratory, and imaging findings can help predict mortality in patients with paraquat poisoning with reasonable accuracy when plasma paraquat concentrations are unavailable. Serial assessment of renal function, liver function, oxygenation, and chest imaging can serve as practical prognostic indicators that are widely available in most hospitals.

## Article #3:

Devlin LA, Babineau DC, Merhar SL, et al. Symptom-Based Dosing for Neonatal Opioid Withdrawal: The OPTimize NOW Randomized Clinical Trial. JAMA 2026;335(24):2114-2125. https://doi.org/10.1001/jama.2026.5782.

### Background

Management of neonatal opioid withdrawal syndrome (NOWS) is guided by both the Eat, Sleep, Console (ESC) approach and the Finnegan score. Infants who do not adequately respond to first-line nonpharmacologic care are treated pharmacologically. This study compares a newer symptom-based opioid dosing approach with the traditional scheduled opioid taper approach.

### Research Question

Compared to a scheduled opioid taper approach, does a symptom-based opioid dosing regimen decrease the time from birth to medical readiness for discharge compared to ESC management for infants with NOWS?

### Methods

A multicenter, stratified, cluster randomized clinical trial was conducted at 23 neonatal intensive care units in the US. Prior to study inception, 15 NICUs used an ESC approach and 8 used a Finnegan-based care model. Infants at least 36 weeks gestation at birth at risk for pharmacologic NOWS treatment were included

Participant sites were randomized to one of two treatment sequences: symptom-based dosing followed by a scheduled opioid taper using the site’s routine scheduled tapering algorithm, or a scheduled opioid taper followed by symptom-based dosing. During the symptom-based dosing approach, infants received a single opioid dose and were monitored for persistent or worsening withdrawal requiring further opioid dosing. Infants with persistently severe opioid withdrawal in the symptom-based group were transitioned to a scheduled opioid taper.

### Results

626 infants were enrolled; 383 infants in the ESC cohort were included in the primary analysis: 189 infants received symptom-based opioid treatment and 194 received scheduled opioid tapers. Mean time to medical readiness to discharge was significantly less in the symptom-based treatment group compared to the scheduled taper group (9.2 vs 11.6 days, adjusted mean ratio 0.79 [95% CI, 0.65-0.96]). Secondary outcomes including length of stay and likelihood of receiving additional pharmacologic treatment were not significantly different between the symptom-based or scheduled taper groups. Of the symptom-based pharmacologically treated infants, 35% had persistently severe withdrawal necessitating scheduled opioids. Both inpatient and post-discharge safety events were rare.

### Conclusion

Compared to a scheduled opioid taper, symptom-based opioid dosing decreased the time to medical readiness to discharge in infants treated with the ESC-based approach for NOWS.

### Critique

This was a large and methodologically sophisticated pragmatic clinical trial. Strengths include randomization stratified by the treatment paradigm already in use by the site, standardized assessments, and frequent patient reassessment. Study limitations arose from the variability of treatment protocols between sites, variability in the use of adjunctive medications (eg, phenobarbital, clonidine), and incomplete ability to assess outcomes after hospital discharge.

### Implication for Toxicologists

Symptom-based opioid dosing for NOWS is a reasonable approach for pharmacologic treatment which, in this study, shortened hospital length of stay by about 2½ days. Some infants on the as-needed protocol may experience persistently severe opioid withdrawal, ultimately needing scheduled opioids. The thoughtful editorial on this study in the same issue of JAMA is worth reading: https://doi.org/10.1001/jama.2026.6412.

## Article #4:

Simmons R, Ubani C, Garvan G, et al. Retrospective Comparison of Empiric Antivenom vs. Expectant Treatment for Eastern Coral Snakebites. West J Emerg Med 2025;27(1):167-176. https://doi.org/10.5811/westjem.45709.

### Background

There is limited research as to whether empiric administration of North American Coral Snake Antivenom (NACSA) vs a wait-and-see approach is superior in the treatment of asymptomatic patients bitten by eastern coral snakes (Micrurus fulvius). NACSA is an older whole immunoglobulin antivenom, for which the risk of an acute hypersensitivity reaction must be weighed against the potential benefit of preventing neuromuscular paralysis that NACSA cannot reverse.

### Research Question

Are there differences in outcome in a wait-and-see approach compared to empiric antivenom treatment in initially asymptomatic patients with an eastern coral snake bite?

### Methods

This was a retrospective observational study of eastern coral snake exposures reported to the Florida Poison Information Network between 1998 – 2021. Included cases were patients bitten by a coral snake who were asymptomatic (other than pain or an allergic reaction to the venom) and monitored for over 24 hours in a health care facility. Four independent non-blinded reviewers collected variables from the network’s database, including patient demographics, bite details (eg, means of snake identification, latching, anatomical bite location), clinical effects, treatment provided, clinical outcomes, and adverse effects of NACSA. Patients in the “empiric” group were those who received antivenom at the time of initial presentation to the emergency department, before any symptoms of envenomation developed. Patients in the “wait-and-see” group were observed with a plan to administer antivenom if signs or symptoms of envenomation appeared. Multivariate regression models controlled for variables such as demographics and antivenom shortages.

### Results

Out of 1,082 identified cases, 301 met inclusion criteria: 171 cases in the empiric group, and 130 in the wait-and-see group. Twenty patients (15.4%) in the wait-and-see group developed systemic symptoms resulting in NACSA treatment. A total of 60.2% of patients in the empiric antivenom and 72.3% of patients in the wait-and-see group remained free of systemic symptoms. The likelihood of ICU admission was higher in the empiric group (75.2%) than in the wait-and-see group (56.8%; P = 0.05), however there was no difference in the median hospital length of stay (1 day vs 1 day; P = 0.19) or need for intubation (1.2% vs 0.8%; P = 0.63). Of the 191 patients receiving NACSA, 38 (19.9%) developed adverse effects, most of which were minor. Anaphylaxis occurred in 4.2% of patients receiving NACSA, and two (1.0%) antivenom-treated patients required intubation for adverse effects of antivenom.

### Conclusion

Relative to a wait-and-see approach, empiric NACSA administration was associated with a higher likelihood of ICU admission, with no statistical difference in ICU length of stay, hospital length of stay, or the incidence of intubation.

### Critique

Eastern coral snake bites are relatively uncommon (1,082 potential exposures in Florida reviewed for this observational study in 23 years) and most patients do not develop systemic toxicity, limiting key statistical analyses in this study. This study is subject to the limitations of all poison center studies, including reporting bias, missing data, and inability to validate clinical findings. Like all observational studies, this study is vulnerable to bias by indication and unmeasured confounding: patients receiving empiric antivenom may have also had clinical features (eg, latching) suggesting higher risk. These data cannot be generalized to different coral snake species (eg, *Micrurus tener* or *Micruroides euryxanthus*).

### Implication for Toxicologists

Within its limitations, this study supports observation over empiric antivenom use in asymptomatic patients bitten by eastern coral snakes, possibly reducing harm and cost.

## Article #5:

Kliewer RS, Kemp JM, Cliburn KD, et al. Determination of Mitragynine Concentrations in Medicolegal Death Investigations at the Oklahoma Office of the Chief Medical Examiner from 2022 to 2023. J Forensic Sci 2026; ePub May 28. https://doi.org/10.1111/1556-4029.70370.

### Background

Kratom (*Mitragyna speciosa*) and its metabolite 7-hydroxymitragynine (7-OH) are partial agonists at the μ-opioid receptor and competitive antagonists at the κ-opioid and δ-opioid receptors. While both the plant and synthetic 7-OH are historically legal and widely available in the US, in July 2026, the US Drug Enforcement Administration published Notices of Intent to move products containing more than 0.05% 7-OH or any amount of three 7-OH analogs to schedule 1 under the Controlled Substances Act.

### Research Question

Can postmortem mitragynine concentrations in central blood, peripheral blood, and liver be used reliably during medicolegal death investigations, and does mitragynine demonstrate significant postmortem redistribution?

### Methods

Investigators retrospectively reviewed medicolegal death investigations performed at the Oklahoma Office of the Chief Medical Examiner in 2022 and 2023. Mitragynine was quantified using a validated LC-MS/MS assay in central blood, femoral blood, and liver tissue, while 7-OH was assessed qualitatively. The study population was decedents in whom mitraginine was detected (limit of quantification: 50 ng/mL). Central-to-peripheral (C/P) and liver-to-peripheral (L/P) concentration ratios were calculated to evaluate postmortem redistribution.

### Results

Among 12,623 medicolegal cases, 65 contained detectable mitragynine (median age: 40 years; 71% male; 93% non-Hispanic white), and 28 cases had paired specimens available for analysis of postmortem redistribution. Among decedents in whom mitraginine was detected, the median mitraginine concentrations were 550 ng/mL in heart blood, 570 ng/mL in femoral blood, and 950 ng/g in liver tissue. The mean central-to-peripheral serum concentration ratio (C/P) ratio was 1.01 and the mean liver to peripheral blood (L/P) concentration ratio was 3.45, with neither ratio correlating with the estimated postmortem interval. These findings suggest minimal postmortem redistribution of mitragynine, although significant variability was observed (C/P range: 0.31 – 1.86; L/P range: 0.86 – 9.70).

Most deaths involved multiple substances, most commonly fentanyl (detected in 54%), and 79% of death certificates listed mitragynine as a contributing cause rather than the sole cause of death. Only four cases contained detectable 7-OH.

### Conclusion

Mitragynine demonstrates minimal postmortem redistribution, making peripheral blood concentrations relatively reliable for forensic interpretation. These data provide useful reference ranges for forensic pathologists investigating deaths involving kratom products.

### Critique

Most deaths involved polysubstance exposure, particularly illicit fentanyl, making it difficult to determine the independent contribution of mitragynine to fatality. Environmental conditions before recovery, the duration between death and specimen collection, and the timing of kratom ingestion were largely unknown. 7-OH was only measured qualitatively and was found in only a small number of samples, limiting assessment regarding the role of 7-OH. Assays for other potentially concerning 7-OH analogs were not reported.

### Implication for Toxicologists

This study provides potentially helpful reference data for forensic toxicologists and medical examiners interpreting postmortem mitragynine concentrations. It is difficult to evaluate the relative contribution of kratom, other substances, and other causes to these deaths. The finding of minimal postmortem redistribution supports a greater confidence in peripheral blood measurements during cause-of-death investigations.

## Declarations

The reviews in this article have not previously been published in any abstracts or presented in any meeting.

